# Predicting Depression, Anxiety, and Their Comorbidity among Patients with Breast Cancer in China Using Machine Learning: A Multisite Cross-Sectional Study

**DOI:** 10.1155/2024/3923160

**Published:** 2024-06-21

**Authors:** Shu Li, Jing Shi, Chunyu Shao, Kristin K. Sznajder, Hui Wu, Xiaoshi Yang

**Affiliations:** ^1^China Medical University College of Health Management, Shenyang 110122, Liaoning Province, China; ^2^The First Affiliated Hospital of China Medical University, Shenyang 110122, Liaoning Province, China; ^3^Pennsylvania State University College of Medicine, 500 University Drive, Hershey 17033, PA, USA

## Abstract

Depression and anxiety are highly prevalent among patients with breast cancer. We tested the capacity of personal resources (psychological resilience, social support, and process of recovery) for predicting depression, anxiety, and comorbid depression and anxiety (CDA) among such patients using machine learning (ML). We conducted a cross-sectional survey in Liaoning Province, China, including questions about demographics, COVID-19′s impact, and personal resources (707 valid responses). In the training set, we used Lasso logistic regression to establish personal resource models. Subsequently, we used six ML methods and a tenfold cross-validation strategy to establish models combining personal resources, demographics, and COVID-19 impacts. Findings indicate that in total, 21.9%, 35.1%, and 14.7% of participants showed depression, anxiety, and CDA, respectively. Loneliness, vitality, mental health, bodily pain, and self-control predicted depression, anxiety, and CDA. Furthermore, general health predicted depression, and physical function predicted anxiety. Demographic and COVID-19 models were far less predictive than personal resource models (0.505–0.629 vs. 0.826–0.869). Among combined models, the support vector machine model achieved the best prediction (AUC: 0.832–0.873), which was slightly better than the personal resource models. Personal resources features with ML and personal resources can help predict depression, anxiety, and CDA in patients with breast cancer. Accordingly, interventions should target loneliness, bodily pain, vitality, mental health, and self-control.

## 1. Introduction

Malignant breast cancer tumors are a major issue affecting women's health worldwide; in 2020, they surpassed lung tumors to become the malignant tumor with the highest global incidence rate [1]. Breast cancer, as a major life event, hugely impacts patients' physical and mental health; depression, anxiety, panic, and posttraumatic stress symptoms are common. This was especially true during the COVID-19 pandemic. However, health professionals often overlook patients' mental health status [2].

Depression and anxiety are the most common psychological conditions among patients with breast cancer; their prevalence rates range from 9.4 to 66.1% and 17.9 to 33.3%, respectively [3]. These conditions often appear together as comorbidities, which was first proposed by Feinstein [4] Lu of Yale University in 1970. Recently, comorbid depression and anxiety (CDA) has become a hot topic in the psychiatry field owing to its drastic impact on treatment adherence, survival rates, and life satisfaction for patients with breast cancer.

The factors that contribute to the development of depression and anxiety in patients with breast cancer are complex and multifaceted. While previous research has predominantly concentrated on clinical factors as predictors or demographic features and physical symptoms such as fatigue, weakness, pain, and insomnia [5, 6, 7, 8], a multitude of factors beyond the cancer itself likely contribute. For example, demographic characteristics and personal resource factors such as social support, loneliness, sleep quality, and quality of life (QOL) have been associated with an increased risk of depression and anxiety in patients with breast cancer [9]. A comprehensive perspective of the associated factors of depression and anxiety was adopted in this study, and this study, based on the positive psychology theory [10], considered not only demographic and negative psychological variables as well as physical symptoms but also a range of positive psychological resource feature variables. In contrast to traditional psychology, which tends to focus on negative aspects such as mental illness and disorder, positive psychology emphasizes the positive aspects of human experience, focusing on how happiness, well-being, and flourishing can lower the prevalence of depression and anxiety [10]. Positive psychological capacities such as self-efficacy, resilience, recovery experience, self-control, and hope have been found to be negatively associated with the development of depression and anxiety [11, 12, 13, 14, 15, 16, 17]. Among these, self-control refers to the psychological ability of an individual to regulate their own behavior, emotions, and thoughts to achieve long-term goals, resist temptations or impulses, and conform to social norms and values; it has been considered an important aspect of self-regulation, which is crucial for achieving success in various domains of life such as academic achievement, work performance, and psychological health [11]. Considering the broad aim of improving the psychological health of patients with breast cancer, our research explores a range of personal resource factors, including resilience, recovery experience, QOL, and posttraumatic growth, as predictors of depression, anxiety, and CDA.

Machine learning (ML) is a promising tool that has been increasingly applied in the field of cancer research to predict patient outcomes and identify risk factors for various cancer-related problems, including aspects of psychological distress such as depression and anxiety [18]. ML algorithms can analyze large datasets and identify predictors and relationships that may not be apparent using traditional statistical methods such as logistic regression analysis [19]. Some studies have used ML to predict depression or anxiety of breast cancer patients [6, 7]; however, there is no study using multiple ML methods to simultaneously predict depression, anxiety, and CDA.

In line with the quality standards of transparent reporting of multivariate prediction models for individual prognosis or diagnosis (TRIPOD) reporting guidelines [20], this study aimed to use ML algorithms to evaluate the prevalence of depression, anxiety, and CDA among patients with breast cancer during the COVID-19 pandemic and find relevant predictors. The findings will provide a theoretical and evidence-based foundation for efforts to improve the psychological health and well-being of patients with breast cancer; they may also assist in identifying patients at risk of developing depression and anxiety and suggest targeted interventions to minimize the risk of these psychological problems.

## 2. Methods

### 2.1. Participants

This cross-sectional study was conducted in tertiary hospitals in seven cities of Liaoning Province (nine hospitals in total) from January 10 to April 30, 2021. According to the distribution traits of the breast cancer population, combined with regional characteristics and economic factors in Liaoning Province, the stratified sampling was adopted in this study. Nine tertiary hospitals (two tertiary hospitals in Shenyang and two tertiary hospitals in Dalian and one tertiary hospital in each city of Jinzhou, Panjin, Fushun, Chaoyang, and Huludao) were randomly selected from seven cities of Liaoning Province. The patients who met the inclusion criteria were continuously selected from each hospital. The inclusion criteria were (1) aged 18 years and above; (2) diagnosed with breast cancer; (3) voluntarily agreed to participate in the survey; and (4) without lifetime diagnosis of any psychological disorder. All the participants were well informed of the purposes and contents of this study. Informed consent was obtained from the participants ahead of the survey. Finally, around 800 patients with breast cancer were recruited. Face-to-face questionnaires were conducted with patients to evaluate their mental health status. After excluding illogical answers, invalid responses, and responses with missing values, the response rate was 88.38% (707 survey responses). This study was conducted following the Helsinki Declaration (1989). The study protocols were approved by the Ethics Committee at China Medical University.

### 2.2. Measures

The questionnaire consisted of two parts: general information and psychological health evaluation. The general information part consisted of demographic characteristics and information relating to COVID-19 (COVID-19 impact). Demographic characteristics included age, marital status, education, income, and chronic disease. COVID-19 impact factors included whether the COVID-19 pandemic affected how treatment was conducted (impact on treatment way (IOTW)), whether treatment was delayed or interrupted by COVID-19 (delay or interruption of treatment (DIOT)), whether the COVID-19 outbreak lengthened the period from diagnosis to hospitalization (extended time (ET)), how the COVID-19 outbreak affected patients' family income (impact on income (IOI)), how COVID-19 affected patients' daily lives (impact on daily life (IODL)), and how COVID-19 affected how patients' families provided care (impact on caregiving (IOC)).

The psychological health part of the questionnaire used items from the Self-Rating Anxiety Scale [21] (Cronbach *α* coefficient = 0.767), Patient Health Questionnaire-9 [22] (Cronbach *α* coefficient = 0.768), Perceived Social Support Scale [23] (Cronbach *α* coefficient = 0.964), Life Orientation Test-Revised [24] (Cronbach *α* coefficient = 0.702), Medical Outcomes Study 36-Item Short Form [25] (Cronbach *α* coefficient = 0.900), Adult Hope Scale [26] (Cronbach *α* coefficient = 0.943), Self-Control Scale [27] (Cronbach *α* coefficient = 0.726), General Self-Efficacy Scale [28] (Cronbach *α* coefficient = 0.946), Posttraumatic Growth Inventory [29] (Cronbach *α* coefficient = 0.973), Ego Resilience Scale [30] (Cronbach *α* coefficient = 0.926), Loneliness University of California at Los Angeles [31] (Cronbach *α* coefficient = 0.702), Questionnaire on Process of Recovery [32] (Cronbach *α* coefficient = 0.968), Chinese version of General Self-efficacy Scale [33] (Cronbach *α* coefficient = 0.928), and Brief Self-Control Scale (Cronbach *α* coefficient = 0.906) [27] (Cronbach *α* coefficient = 0.878).

Through these questionnaires, 27 personal resource features were obtained. Process of recovery was categorized into three dimensions: self-empowerment, interpersonal relationship, and building life. The posttraumatic growth had five dimensions: new possibilities, personal strength, spiritual change, relating to others, and appreciation of life. Quality of life had eight dimensions: physical health (RP), bodily pain (BP), general health perceptions (GH), role limitations due to function (RF) vitality (VT), social functioning (SF), role limitations due to emotional (RE), and mental health (MH) as well as physical component summary (PCS) and mental component summary (MCS). The remaining features contained only one total score. In addition to these subfeatures, a total score was calculated for each class.

Detailed instructions of all features are shown in *Supplementary 1* of the Supplementary material S1.

### 2.3. Data Preparation, Model Establishment, and Performance Evaluation

**Figure 1:**
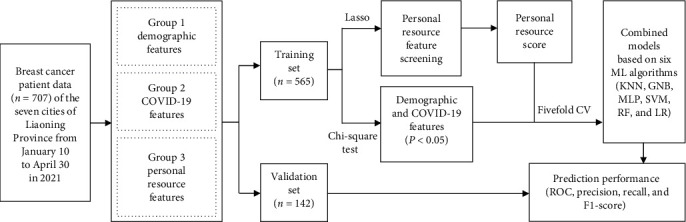
Detailed flowchart of this study.

All valid survey data were randomly divided into a training set (*n* = 565) and a validation set (*n* = 142) in an 8 : 2 ratio. Considering the relatively severe data imbalance between patients with and without depression and patients with and without CDA, we used the Borderline-SMOTE algorithm to oversample the training set data. This can enable the model to better learn features from the minority categories, thereby improving the model's generalization ability and accuracy [34]. A detailed flowchart of the study procedures is shown in Figure 1.

In this study, demographic models, COVID-19 impact models, personal resource models, and combined models were established to predict depression, anxiety, and CDA among patients with breast cancer. Before establishing the models, the features were normalized by the *z*-score.

Firstly, the *chi*-square test was used to analyze and screen demographic and COVID-19 impact features. *P* < 0.05 was considered to denote a significant difference. Demographic models and COVID-19 impact models were established using stepwise logistic regression methods based on statistically significant features in the training set.

Secondly, in the training set, least absolute shrinkage and selection operator (Lasso) logistic regression were used to screen personal resource features and establish personal resource models for identifying depression, anxiety, and CDA. Lasso logistic regression can effectively extract important variables from numerous variables, thereby reducing model complexity and avoiding overfitting. It also has the advantage of being able to solve the multicollinearity problem between variables while maintaining good interpretability [35]. A tenfold cross-validation strategy was employed to tune hyperparameter C (inverse of the regularization strength) to determine the magnitude of penalization. The personal resource score of each patient with breast cancer was calculated using the linear combination of features with nonzero coefficients and their corresponding coefficients.

Subsequently, combining significant demographic features (*P* < 0.05), COVID-19 impact features (*P* < 0.05), and personal resource scores, six ML algorithms (*k*-nearest neighbors (KNN), Gaussian naive Bayes (GNB), multilayer perceptron (MLP), support vector machine (SVM), random forest (RF), and stepwise logistic regression (LR)) and a tenfold cross-validation strategy were used to establish combined models for predicting depression, anxiety, and CDA among patients with breast cancer. Some interacting hyperparameters were adjusted to achieve optimal model prediction performance. Detailed instructions and hyperparameter adjustment results are shown in *Supplementary 2*.

Finally, we evaluated and compared the performance of each model (demographic, COVID-19 impact, personal resources, and combined models) on the validation set. Furthermore, DeLong test was used to compare the receiver operating characteristic (ROC) of each category model. DeLong et al. [36] test is a commonly used hypothesis testing method that compares the performance of two classifiers based on the difference in the area under the curve (AUC). *P* < 0.05 was considered to denote a significant difference between the two ROC curves.

### 2.4. Data Analysis

In this study, all methods were implemented using Python 3.7. The stepwise LR algorithm employed the Statsmodes package, while the other five ML and LASSO methods employed the sklearn package. The Borderline-SMOTE algorithm employed the Imblearn package, and the chi-square test employed the Scipy package.

## 3. Results

### 3.1. Demographic Characteristics and COVID-19 Impact

Among the 707 patients with breast cancer, 248, 155, and 104 had depression, anxiety, and CDA, respectively. Detailed demographic characteristics and COVID-19 impact factors are shown in Tables 1, 2, and 3. In the training set, compared with NO depression, depression demonstrated a statistically significant difference based on age, marital status, IOTW, ET, and IOC. Anxiety demonstrated a statistically significant difference compared to NO anxiety in education level, IOTW, DIOT, ET, IOI, IODL, and IOC. DIOT, ET, IODL, and IOC showed statistically significant differences between CDA and no CDA; however, no demographic features were significantly related.

Demographic models and COVID-19 impact models used to predict depression, anxiety, and CDA were established based on the above significant features, respectively. Notably, because of the absence of significant demographic features for CDA in the training set, a demographic model for predicting CDA was not established.

The findings suggest that patients with breast cancer with lower education levels were more likely to have anxiety, while older patients with unstable marriages were more likely to have depression. Furthermore, the changes brought about by COVID-19 (relating to both medical treatment and life in general) created anxiety and depression in patients with breast cancer to a certain extent. Overall, COVID-19 had a greater impact on anxiety.

### 3.2. Personal Resource Model Construction

**Figure 2:**
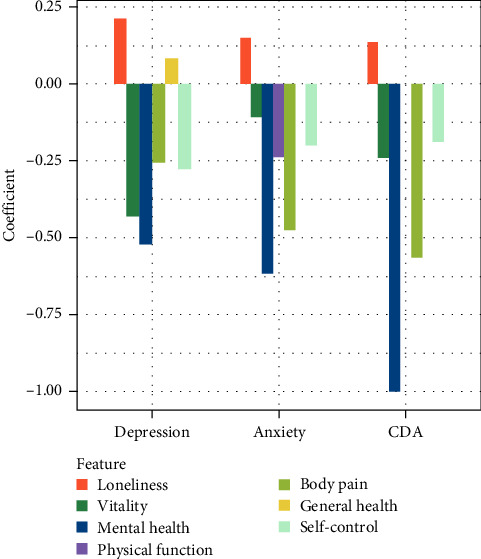
Personal resource features and coefficients of Lasso logistic regression selection. Abbreviation: comorbid depression anxiety (CAD).

Regarding depression, anxiety, and CDA, when the optimal C-values were set to 0.0451, 0.0785, and 0.0331, Lasso logistic regression selected six, six, and five important features, respectively, to construct three personal resource models. The Lasso regulation profile is shown in Supplementary material S3 (see *Supplementary 3*).  Figure 2 presents the features and their coefficients in the three personal resource models. The larger the absolute value of the coefficient, the more important the corresponding feature is in predicting the dependent variables. The positive or negative coefficients indicate a positive or negative correlation with the dependent variable. The results indicate that although the three personal resource models include five of the same features, the contribution of each feature is different in each model. The most predictive factors for anxiety, depression, and CDA were loneliness, vitality, mental health, bodily pain, and self-control. In addition, general health significantly predicted depression and physical function predicted anxiety. For the identification of depression, anxiety, and CDA, AUCs of the three personal resource models on the validation set were 0.869, 0.826, and 0.862, respectively.

### 3.3. Combined Model Construction and Performance Evaluation

**Figure 3:**
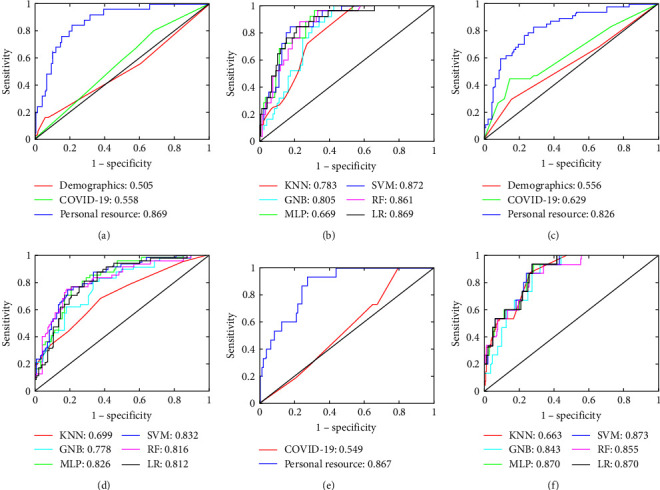
ROC of demographic, COVID-19 impact, personal resource, and combined models on the validation set. (a), (b) ROC of the demographic, COVID-19 impact, personal resource, and six combined ML models in predicting depression, respectively. (c), (d) ROC of the demographic, COVID-19 impact, personal resource, and six combined ML models in predicting anxiety, respectively. (e), (f) ROC of the COVID-19 impact, personal resource, and six combined ML models in CDA, respectively.

The ROCs of the demographic, COVID-19 impact, personal resources, and combined models on the validation set are shown in Figure 3, and the evaluation indicators are shown in Table 4. The demographic and COVID-19 impact models predicted poorly; the personal resource model showed much better prediction and achieved good results in identifying depression, anxiety, and CDA.

The combined model based on the SVM algorithm achieved the highest AUC in identifying depression (0.872), anxiety (0.832), and CDA (0.873), and overall performance was slightly improved compared to the personal resource models. In CDA, the MLP and LR combined models also showed slight improvements compared to the personal resource model.

DeLong test results indicated that in anxiety, there was a significant difference in ROC between the personal resource model and the demographic and COVID-19 models (*P* < 0.001); except for KNN and GNB, there were significant differences in ROC between the other combined models and the demographic model (range: *P* < 0.001 to *P*=0.026). In depression, there was a significant difference in ROC between the personal resource model and the demographic and COVID-19 models (*P* < 0.001); except for KNN, there were significant differences in ROC between other combined models and the demographic model (range: *P* < 0.001 to *P*=0.003). In CDA, there were significant differences in ROC between the personal resource model and the COVID-19 model, as well as between each combination model and the COVID-19 model (range: *P* < 0.001 to *P*=0.002).

It is thus clear that ML algorithms can help identify psychological distress among patients with breast cancer. Overall, personal resource features are the most effective predictors of anxiety, depression, and CDA in patients with breast cancer.

## 4. Discussion

Our study is the first to use ML algorithms to predict depression, anxiety, and CDA in patients with breast cancer in Northeast China. Compared to traditional logistic regression, the use of ML algorithms can address the issue of multicollinearity among variables and identify more effective predictors while considering the relationships between multiple variables. We not only identified important factors predicting anxiety, depression, and CDA but also highlighted the contribution of each feature in the ML model, which may provide a basis for effective intervention measures to alleviate these psychological difficulties among patients with breast cancer.

Psychological problems such as depression and anxiety during treatment for breast cancer can affect treatment outcomes. Therefore, constructing a predictive model for anxiety and depression in patients with breast cancer can help doctors better distinguish patients with a high risk of psychological problems. This can help doctors improve patients' mental health status and enhance their overall life satisfaction. Research has shown that approximately half of cancer patients experience depression and anxiety, which can reduce long-term cancer survival rates by 10%–20% [37]. We found that patients with breast cancer face severe symptoms of depression, anxiety, and CDA; their prevalence rates among our sample were 21.9%, 35.1%, and 14.7%, respectively. Depression was more prevalent in our sample than that reported in a study conducted in Taiwan (6.92%). Furthermore, anxiety was much higher prevalent than in the Taiwanese study (15.9%) [38] and in an American study during the COVID-19 pandemic (29.1%) [39]. However, the rate of CDA was much lower than that in a Ghanaian study (29.4%) [40].

The previous studies used ML to predict depression among patients with breast cancer [7, 41]; however, they only included patients' demographic characteristics or prescription NSAIDs as the leading predictors of depression. Our research also included the influence of COVID-19 and personal resources. We also used a variety of ML methods to establish multiple models to identify depression, anxiety, and CDA among patients with breast cancer, with the highest AUCs of 0.872, 0.832, and 0.873, respectively. In this study, Lasso effectively screened aspects of personal resources. Lasso has high data applicability, especially in dealing with datasets with multiple independent variables and can be applied in studies involving complex features such as omics [42]. Furthermore, Lasso balances the accuracy of predictions with the interpretability of the model. In this study, our models not only screened important personal resource features for predicting depression, anxiety, and CDA but also elucidated the contribution and positive/negative correlations of each feature in the prediction model. Among the various combined models, we found that SVM had the best predictive performance and MLP and LR had good predictive performance, while KNN, GNB, and RF had unstable predictive performance. Therefore, we suggest that SVM, MLP, and LR algorithms should be tried first in psychological research examining patients with breast cancer. After model testing, our most important finding is that personal resources are the best predictors when identifying depression, anxiety, and CDA among patients with breast cancer.

Among the QOL variables, mental health—which mainly assessed individuals' psychological well-being—was the strongest predictor of depression, anxiety, and CDA, followed by bodily pain. Hence, the lower an individual's psychological health and the more severe their symptoms of bodily pain, the more severe the symptoms of depression, anxiety, and CDA will be. These findings have important implications for the development of effective psychological interventions, which should target the modifiable factors that are most predictive of psychological well-being among patients with breast cancer.

Previous research has shown that chronic bodily pain not only causes physical impairments but also increases the odds of adverse emotions such as depression and anxiety [43]. Bodily pain may lead to the onset of depression and anxiety, and when experienced for an extended period, it may cause individuals to feel fatigued, powerless, and helpless, thereby increasing their susceptibility to developing depression and anxiety. Further research is needed to clarify the relationship between pain and depression and anxiety; targeting physical pain may be a potential intervention strategy for alleviating depression and anxiety in patients with breast cancer [44].

Among the other indicators, we found that the QOL indicator of vitality had a negative predictive effect on depression, anxiety, and CDA. Our results also revealed that loneliness positively predicted depression, anxiety, and CDA, which was consistent with previous research [45]. Loneliness—referring to a subjective psychological experience in which individuals suffer from a lack of connections with others—is a common issue and is closely related to depression and anxiety [46]. Feelings of loneliness may lead to unfulfilled emotional needs and result in negative emotions, decreased self-esteem, self-doubt, and self-negation, thereby increasing the risk of depression and anxiety [47]. During the COVID-19 pandemic, an increase in loneliness among patients with breast cancer was found to be significantly correlated with a worsening of depression and anxiety symptoms [48]. Our study supports these results, showing that patients with breast cancer reporting higher levels of loneliness were more likely to experience psychological disorders such as depression, potentially jeopardizing their willingness to persist with treatment and improving their health condition [49].

In addition, this study found that self-control—referring to an individual's ability to influence, regulate, and control their own psychological, behavioral, and physiological processes—was inversely associated with depression, anxiety, and CDA. This is consistent with previous findings [11, 12, 13, 14, 15, 16, 17], which indicated that self-control had a predictive impact on depression, anxiety, and CDA [50]. Another study found that self-control was negatively correlated with negative emotions such as depression and anxiety [51]. Individuals with high self-control tend to exhibit superior performance in controlling their thoughts, regulating their emotions, and inhibiting impulsive behaviors. They also tend to have a greater sense of control over their lives and are more likely to actively seek solutions when facing difficulties. Conversely, those with low self-control might be more passive and prone to experiencing symptoms of depression [52] because they find it more difficult to regulate their emotions and thoughts and are thus more likely to get caught in a cycle of negative emotions and thoughts, affecting their mental health, and increasing depression and anxiety. This study thus highlights self-control as a potential intervention target for alleviating depression and anxiety symptoms in patients with breast cancer.

Our study found five factors that can help identify CDA among patients with breast cancer, indicating significant overlaps between these conditions. We also found two features that predicted depression and anxiety separately. Specifically, general health—which measures an individual's assessment of their own health status and general well-being—was a significant positive predictor of depression, and physical function, which measures whether an individual's health condition interferes with their normal physiological activities, negatively predicted anxiety. The higher the physical function score (i.e., the healthier the physiological functioning of the body), the less likely patients were to suffer from anxiety symptoms. These findings indicate that depression and anxiety are two distinct psychological problems. Thus, interventions should be targeted at general health to reduce depression and physical function to attenuate anxiety.

In addition, although the COVID-19 impact models showed a limited ability to predict depression, anxiety, and CDA, COVID-19 had some explanatory impact. For example, delay or interruption of treatment and impact on income were risk factors for anxiety, and impact on daily life was a risk factor for CDA. Hence, delayed or interrupted treatment and negative impacts on income owing to COVID-19 seemed to make patients more worried and anxious about the disease treatment. Psychological difficulties among patients with breast cancer may be caused by the physiological discomfort brought about by both breast cancer and its treatment, which results in feelings of anxiety. Furthermore, the diagnosis and treatment of breast cancer may impose a heavy burden on the social and family lives of patients, including job loss, financial stress, and family conflicts, which can also lead to the development of anxiety symptoms [53, 54]. The impact of the COVID-19 pandemic exacerbated these aspects; the upheaval to everyday life it produced may have triggered more physical and physiological discomfort, including pain, fatigue, nausea, and vomiting, further impacting patients' mental health status, and increasing the prevalence of comorbid depression and anxiety.

## 5. Conclusion

Patients with breast cancer in China exhibited high rates of depression and anxiety and a low rate of CDA during the COVID-19 pandemic. Methodologically, ML is a powerful tool to predict depression, anxiety, and CDA among patients with breast cancer; it can successfully identify relevant predictors. Finally, the results of this study have implications for psychological interventions among patients with breast cancer. Namely, we recommend targeted interventions to manage patients' loneliness, improve their physical and mental symptoms, and enhance their self-control, which can reduce the prevalence of depression, anxiety, and CDA.

## Figures and Tables

**Table 1:** Demographics and COVID-19 impact features of depression among breast cancer patients.

Demographics and COVID-19 features	Total (%)	Training set			Validation set		
		Depression (%)	No depression (%)	*P* value	Depression (%)	No depression (%)	*P* value
	707 (100)	130 (23.00)	435 (77.00)	—	25 (17.61)	117 (82.39)	—
Age (years)	—	—	—	**0.023** ***⁣* ^*∗*^**	—	—	—
<55	334 (47.24)	50 (38.46)	219 (50.34)	—	13 (52.00)	52 (44.44)	0.640
≥55	373 (52.76)	80 (61.54)	216 (49.66)	—	12 (48.00)	65 (55.56)	—
Marital status	—	—	—	**0.020*⁣*^*∗*^**	—	—	—
Unmarried	24 (3.40)	7 (5.38)	13 (2.99)	—	1 (4.00)	3 (2.56)	0.398
Married	645 (91.23)	111 (85.38)	405 (93.10)	—	21 (84.00)	108 (92.31)	—
Others	38 (5.37)	12 (9.23)	17 (3.91)	—	3 (12.00)	6 (5.13)	—
Education	—	—	—	0.120	—	—	—
Primary school and bellow	307 (43.42)	62 (47.69)	183 (42.07)	—	15 (60.00)	47 (40.17)	0.179
Senior high school	201 (28.43)	43 (33.08)	129 (29.66)	—	3 (12.00)	26 (22.22)	—
College and above	199 (28.15)	25 (19.23)	123 (28.28)	—	7 (28.00)	44 (37.61)	—
Income	—	—	—	0.578	—	—	—
<3,000	318 (44.98)	62 (47.69)	186 (42.76)	—	15 (60.00)	55 (47.01)	0.079
3,000–6,000	261 (36.92)	46 (35.38)	163 (37.47)	—	10 (40.00)	42 (35.90)	—
≥6,000	128 (18.10)	22 (16.92)	86 (19.77)	—	0 (0.00)	20 (17.09)	—
Chronic disease	—	—	—	0.117	—	—	—
Yes	341 (48.23)	73 (56.15)	208 (47.82)	—	14 (56.00)	46 (39.32)	0.190
No	366 (51.77)	57 (43.85)	227 (52.18)	—	11 (44.00)	71 (60.68)	—
IOTW	—	—	—	**0.036** ***⁣* ^*∗*^**	—	—	—
Yes	234 (33.10)	48 (36.92)	117 (26.90)	—	15 (60.00)	54 (46.15)	0.230
No	473 (66.90)	82 (63.08)	318 (73.10)	—	10.0 (40.00)	63 (53.85)	—
DIOT	—	—	—	0.117	—	—	—
Yes	318 (44.98)	73 (56.15)	208 (47.82)	—	6 (24.00)	31 (26.50)	0.994
No	389 (55.02)	57 (43.85)	227 (52.18)	—	19 (76.00)	86 (73.50)	—
ET	—	—	—	**0.010** ***⁣* ^*∗*^**	—	—	—
Yes	206 (29.14)	51 (39.23)	117 (26.90)	—	8 (32.00)	30 (25.64)	0.687
No	501 (70.86)	79 (60.77)	318 (73.10)	—	17 (68.00)	87 (74.36)	—
IOI	—	—	—	0.240	—	—	—
Yes	485 (68.60)	95 (73.08)	292 (67.13)	—	22 (88.00)	76 (64.96)	**0.043** ***⁣* ^*∗*^**
No	222 (31.40)	35 (26.92)	143 (32.87)	—	3 (12.00)	41 (35.04)	—
IODL	—	—	—	0.055	—	—	—
Yes	537 (75.95)	107 (82.31)	320 (73.56)	—	86 (73.50)	24 (96.00)	**0.029** ***⁣* ^*∗*^**
No	170 (24.05)	23 (17.69)	115 (26.44)	—	31 (26.50)	1 (4.00)	—
IOC	—	—	—	**0.001^*∗∗*^**	—	—	—
Yes	477 (67.47)	102 (78.46)	275 (63.22)	—	20 (80.00)	80 (68.38)	0.360
No	230 (32.53)	28 (21.54)	160 (36.78)	—	5 (20.00)	37 (31.62)	—

*⁣* ^*∗*^ *P*  < 0.05, *⁣*^*∗∗*^*P*  < 0.01. Bold values: *P* < 0.05.

**Table 2:** Demographics and COVID-19 features of anxiety among breast cancer patients.

Demographics and COVID-19 features	Total (%)	Training set			Validation set		
		Anxiety (%)	No anxiety (%)	*P*-value	Anxiety (%)	No anxiety (%)	*P*-value
	707 (100)	201 (35.58)	364 (64.42)	—	47 (33.10)	95 (66.90)	—
Age (years)	—	—	—	0.205	—	—	0.151
<55	334 (47.24)	88 (43.78)	181 (49.73)	—	17 (36.17)	48 (50.53)	—
≥55	373 (52.76)	113 (56.22)	183 (50.27)	—	30 (63.83)	47 (49.47)	—
Marital status	—	—	—	0.705	—	—	0.062
Unmarried	24 (3.40)	6 (2.99)	14 (3.85)	—	2 (4.26)	2 (2.11)	—
Married	645 (91.23)	183 (91.04)	333 (91.48)	—	39 (82.98)	90 (94.74)	—
Others	389 (5.37)	12 (5.97)	17 (4.67)	—	6 (12.77)	3 (3.16)	—
Education	—	—	—	**0.019*⁣*^*∗*^**	—	—	0.136
Primary school and bellow	307 (43.42)	92 (45.77)	153 (42.03)	—	17 (36.17)	45 (47.37)	—
Senior high school	201 (28.43)	70 (34.83)	102 (28.02)	—	14 (29.79)	15 (15.79)	—
College and above	199 (28.15)	39 (19.40)	109 (29.95)	—	16 (34.04)	35 (36.84)	—
Income	—	—	—	0.366	—	—	0.708
<3,000	318 (44.98)	84 (41.79)	164 (45.05)	—	24 (51.06)	46 (48.42)	—
3,000–6,000	261 (36.92)	82 (40.80)	127 (34.89)	—	18 (38.30)	34 (35.79)	—
≥6,000	128 (18.10)	35 (17.41)	73 (20.05)	—	5 (10.64)	15 (15.79)	—
Chronic disease	—	—	—	0.134	—	—	0.394
Yes	293 (41.44)	74 (36.82)	159 (43.68)	—	17 (36.17)	43 (45.26)	—
No	414 (58.56)	127 (63.18)	205 (56.32)	—	30 (63.83)	52 (54.74)	—
IOTW	—	—	—	**0.002** ***⁣* ^*∗∗*^**	—	—	**0.017** ***⁣* ^*∗∗*^**
Yes	350 (49.50)	118 (58.71)	163 (44.78)	—	30 (63.83)	39 (41.05)	—
No	357 (50.50)	83 (41.29)	201 (55.22)	—	17 (36.17)	56 (58.95)	—
DIOT	—	—	—	<0.001^*∗∗*^	—	—	0.611
Yes	202 (28.57)	93 (46.27)	72 (19.78)	—	14 (29.79)	23 (24.21)	—
No	505 (71.43)	108 (53.73)	292 (80.22)	—	33 (70.21)	72 (75.79)	—
ET	—	—	—	<0.001^*∗∗*^	—	—	<0.001^*∗∗*^
Yes	206 (29.14)	94 (46.77)	74 (20.33)	—	22 (46.81)	16 (16.84)	—
No	501 (70.86)	107 (53.23)	290 (79.67)	—	25 (53.19)	79 (83.16)	—
IOI	—	—	—	<0.001^*∗∗*^	—	—	0.237
Yes	485 (68.60)	157 (78.11)	230 (63.19)	—	62 (65.26)	36 (76.60)	—
No	222 (31.40)	44 (21.89)	134 (36.81)	—	33 (34.74)	11 (23.40)	—
IODL	—	—	—	**0.003** ***⁣* ^*∗∗*^**	—	—	0.081
Yes	537 (75.95)	167 (83.08)	260 (71.43)	—	41 (87.23)	69 (72.63)	—
No	170 (24.05)	34 (16.92)	104 (28.57)	—	6 (12.77)	26 (27.37)	—
IOC	—	—	—	**<0.001** ***⁣* ^*∗*^**	—	—	0.183
Yes	477 (67.47)	159 (79.10)	218 (59.89)	—	37 (78.72)	63 (66.32)	—
No	230 (32.53)	42 (20.90)	146 (40.11)	—	10 (21.28)	32 (33.68)	—

*⁣* ^*∗*^ *P*  < 0.05, *⁣*^*∗∗*^*P*  < 0.01. Bold values: *P*  < 0.05.

**Table 3:** Demographics and COVID-19 features of CDA among breast cancer patients.

Demographics and COVID-19 features	Total (%)	Training set			Validation set		
		Comorbidity (%)	No comorbidity (%)	*P*-value	Comorbidity (%)	No comorbidity (%)	*P*-value
	707 (100)	89 (15.75)	476 (84.25)	—	15 (10.56)	127 (89.44)	—
Age (years)	—	—	—	0.174	—	—	1.000
<55	334 (47.24)	36 (40.45)	233 (48.95)	—	7 (46.67)	58 (45.67)	—
≥55	373 (52.76)	53 (59.55)	243 (51.05)	—	8 (53.33)	69 (54.33)	—
Marital status	—	—	—	0.445	—	—	**0.041** ***⁣* ^*∗*^**
Unmarried	24 (3.40)	3 (3.37)	17 (3.57)	—	1 (6.67)	3 (2.36)	—
Married	645 (91.23)	79 (88.76)	437 (91.81)	—	11 (73.33)	118 (92.91)	—
Others	38 (5.37)	7 (7.87)	22 (4.62)	—	3 (20.00)	6 (4.72)	—
Education	—	—	—	0.279	—	—	0.399
Primary school and bellow	307 (43.42)	37 (41.57)	208 (43.70)	—	9 (60.00)	53 (41.73)	—
Senior high school	201 (28.43)	33 (37.08)	139 (29.20)	—	2 (13.33)	27 (21.26)	—
College and above	199 (28.14)	19 (21.35)	129 (27.10)	—	4 (26.67)	47 (37.01)	—
Income	—	—	—	0.634	—	—	0.328
<3,000	313 (44.27)	35 (39.33)	213 (44.75)	—	12 (80.00)	53 (41.73)	—
3,000–6,000	239 (33.81)	36 (40.45)	173 (36.34)	—	3 (20.00)	27 (21.26)	—
≥6,000	155 (21.92)	18 (20.22)	90 (18.91)	—	0 (0.00)	47 (37.01)	—
Chronic disease	—	—	—	0.452	—	—	0.232
Yes	293 (41.44)	33 (37.08)	200 (42.02)	—	9 (60.00)	51 (40.16)	—
No	414 (58.56)	56 (62.92)	276 (57.98)	—	6 (40.00)	76 (59.84)	—
IOTW	—	—	—	0.452	—	—	0.232
Yes	293 (41.44)	33 (37.08)	200 (42.02)	—	9 (60.00)	51 (40.16)	—
No	414 (58.56)	56 (62.92)	276 (57.98)	—	6 (40.00)	76 (59.84)	—
DIOT	—	—	—	**0.001** ***⁣* ^*∗*^**	—	—	0.799
Yes	202 (28.57)	39 (43.82)	126 (26.47)	—	3 (20.00)	34 (26.77)	—
No	505 (71.43)	50 (56.18)	350 (73.53)	—	12 (80.00)	93 (73.23)	—
ET	—	—	—	<0.001^*∗∗*^	—	—	0.764
Yes	206 (29.14)	42 (47.19)	126 (26.47)	—	5 (33.33)	33 (25.98)	—
No	501 (70.86)	47 (52.81)	350 (73.53)	—	10 (66.67)	94 (74.02)	—
IOI	—	—	—	0.169	—	—	0.205
Yes	485 (68.60)	67 (75.28)	320 (67.23)	—	13 (86.67)	85 (66.93)	—
No	222 (31.40)	22 (24.72)	156. (32.77)	—	2 (13.33)	42 (33.07)	—
IODL	—	—	—	**0.006** ***⁣* ^*∗*^**	—	—	0.219
Yes	537 (75.95)	78 (87.64)	349 (73.32)	—	14 (93.33)	96 (75.59)	—
No	170 (24.05)	11 (12.36)	127 (26.68)	—	1 (6.67)	31 (24.41)	—
IOC	—	—	—	**0.003** ***⁣* ^*∗*^**	—	—	0.576
Yes	477 (67.47)	72 (80.90)	305 (64.08)	—	12 (80.00)	88 (69.29)	—
No	230 (32.53)	17 (19.10)	171 (35.92)	—	3 (20.00)	39 (30.71)	—

*⁣* ^*∗*^ *P*  < 0.05, *⁣*^*∗∗*^*P*  < 0.01. Bold values: *P* < 0.05.

**Table 4:** The evaluation indicators of demographics, COVID-19, personal resource, and six combined models on the validation set in depression, anxiety, and comorbidity.

Group	Model	Precision	Recall	F1-score	AUC
Depression	Demographics	0.572	0.541	0.544	0.505
	COVID-19 impact	0.412	0.500	0.452	0.558
	Personal resource	0.860	0.761	0.787	0.869
	Combined_KNN	0.778	0.810	0.787	0.783
	Combined_GNB	0.765	0.754	0.759	0.805
	Combined_MLP	0.811	0.838	0.802	0.869
	Combined_SVM	0.818	0.838	0.823	0.872
	Combined_RF	0.826	0.838	0.830	0.861
	Combined_LR	0.822	0.838	0.827	0.869

Anxiety	Demographics	0.448	0.669	0.536	0.556
	COVID-19 impact	0.669	0.596	0.594	0.629
	Personal resource	0.783	0.754	0.760	0.826
	Combined_KNN	0.716	0.725	0.692	0.699
	Combined_GNB	0.746	0.746	0.746	0.778
	Combined_MLP	0.728	0.739	0.724	0.826
	Combined_SVM	0.760	0.768	0.761	0.832
	Combined_RF	0.765	0.768	0.749	0.816
	Combined_LR	0.737	0.746	0.738	0.812

Comorbidity	Demographics	—	—	—	—
	COVID-19 impact	0.447	0.500	0.472	0.549
	Personal resource	0.888	0.768	0.808	0.867
	Combined_KNN	0.881	0.852	0.864	0.863
	Combined_GNB	0.893	0.732	0.782	0.843
	Combined_MLP	0.887	0.845	0.861	0.870
	Combined_SVM	0.878	0.810	0.836	0.873
	Combined_RF	0.875	0.831	0.849	0.855
	Combined_LR	0.880	0.817	0.841	0.870

## Data Availability

The datasets used during the current study are available on reasonable request from the corresponding author (xsyang@cmu.edu.cn).

## Additional Points

*Limitations*. Despite the study's contributions, there are some limitations and areas for improvement. Firstly, the study was cross-sectional and had a limited sample size; hence, causation has not been established. Therefore, sample size and quality of data should be expanded to amplify the representativeness of the study. Secondly, the parameters and algorithms used in the model need further optimization and improvement to enhance their predictive performance and reliability. Furthermore, the clinical characteristics of the patients and practical interventions including actual treatment effects also require further consideration. Future research should look at application scenarios and treatment effects of clinical interventions to further inform research applications. *Code Accessibility*. All consulting questions regarding the study code should be directed at the corresponding author.

## Supplementary Materials

Supplementary 1 Table 1: Detailed instructions regarding all features in this study (see Supplementary Table 1).

Supplementary 2 Table 2: Detailed instructions and hyperparameter adjustment results (see Supplementary Table 2).

Supplementary 3 Lasso regulation profile (see Supplementary Figure 1–3).
