# Supplementary material for: Predicting Depression, Anxiety, and Their Comorbidity among Patients with Breast Cancer in China Using Machine Learning: A Multisite Cross-Sectional Study
Source: Depress Anxiety. 2024 Jun 21;2024:3923160. doi: 10.1155/2024/3923160 (PMC11918714; doi:10.1155/2024/3923160)
Supplement: Supplementary 1 — Table 1: Detailed instructions regarding all features in this study (see Supplementary Table 1). [file 3923160.f1.docx]

**Supplementary material S1**

Table S1 Detailed instructions of all features in this srudy

| **Features group** | **Features name** | **Types of Data** |
| --- | --- | --- |
| Demographics features | Age (years) | nominal |
|  | Marital Status | nominal |
|  | Education | nominal |
|  | Income | nominal |
|  | Chronic disease | nominal |
| COVID-19 impact features | IOTW | nominal |
|  | DIOT | nominal |
|  | ET | nominal |
|  | IOI | nominal |
|  | IODL | nominal |
|  | IOC | nominal |
| personal resource features | Process of Recovery | continuous |
|  | self-empowerment | continuous |
|  | interpersonal relationship | continuous |
|  | building life | continuous |
|  | Posttraumatic Growth | continuous |
|  | new possibilities | continuous |
|  | personal strength | continuous |
|  | spiritual change | continuous |
|  | appreciation of life | continuous |
|  | Relating to Others | continuous |
|  | Quality of life- PCS | continuous |
|  | physical health | continuous |
|  | bodily pain | continuous |
|  | general health perceptions | continuous |
|  | role limitations due to function | continuous |
|  | Quality of life- MCS | continuous |
|  | vitality | continuous |
|  | social functioning | continuous |
|  | role limitations due to emotional | continuous |
|  | mental health | continuous |
|  | Ego Resilience Scale | continuous |
|  | Life Orientation Test-Revised | continuous |
|  | The Chinese version of General Self-efficacy | continuous |
|  | Perceived Social Support Scale | continuous |
|  | Adult Hope Scale | continuous |
|  | Brief Self-Control Scale | continuous |
|  | Loneliness University of California at Los Angles | continuous |
