# Supplementary material for: Predicting Depression, Anxiety, and Their Comorbidity among Patients with Breast Cancer in China Using Machine Learning: A Multisite Cross-Sectional Study
Source: Depress Anxiety. 2024 Jun 21;2024:3923160. doi: 10.1155/2024/3923160 (PMC11918714; doi:10.1155/2024/3923160)
Supplement: Supplementary 2 — Table 2: Detailed instructions and hyperparameter adjustment results (see Supplementary Table 2). [file 3923160.f2.docx]

**Supplementary material S2**

A cross-validation grid search method was applied to find the best hyperparameters of the MLP model，i.e. regularization strength (1e-05, 1e-03, 1e-01, 1e+01, 1e+03), and number of layers and number of weights perlayer ([50,100,50], [10,20,50,100], [100,50,25,10]). The radial basis function was used as a kernel function of the SVM model, and a cross-validation grid search method was applied to find the best hyperparameters of the SVM model γ (from 1e-03 to 1e + 03, number =12) and C (from 1e-04 to 1e + 04, numb er=12). The random search method was used to find the best hyperparameter of the RF model and avoid overfitting, including the number of estimators (from 80 to 120, number =5), the maximum depth of the tree (from 2, 5 or 8), minimum number of samples required to segment nodes (3, 5, or 10), and minimum number of samples per leaf node (5, 10, or 15). Stepwise LR was used to analyse the related features of depression, anxiety, and comorbidities.. The number of neighbours selected in the KNN model are 3,4,6,8,10. The GNB classifier uses the default parameters to construct the model.

The results of tuned hyperparameters show Table S2.

**Table S2** The results of tuned hyperparameters

| **Psychological problem** | **ML method** | **parameters of models** | **Combined models** |
| --- | --- | --- | --- |
| **Depression** | KNN | n_neighbors | 10 |
|  | MLP | Regularization strength | 10 |
|  |  | Hidden layer sizes | [100, 50, 25, 10] |
|  | SVM | C | 1000 |
|  |  | Gamma (γ) | 0.0005 |
|  | RF | Number of estimators | 120 |
|  |  | Max depth of trees | 8 |
|  |  | Min samples split | 10 |
|  |  | Min samples leaf | 10 |
|  |  | Bootstrap method | True |
| **Anxiety** | KNN | n_neighbors | 10 |
|  | MLP | Regularization strength | 10 |
|  |  | Hidden layer sizes | [50, 100, 50] |
|  | SVM | C | 1000 |
|  |  | Gamma (γ) | 0.0001 |
|  | RF | Number of estimators | 90 |
|  |  | Max depth of trees | 8 |
|  |  | Min samples split | 3 |
|  |  | Min samples leaf | 15 |
|  |  | Bootstrap method | True |
| **CDA** | KNN | n_neighbors | 10 |
|  | MLP | Regularization strength | 10 |
|  |  | Hidden layer sizes | [100, 50, 25, 10] |
|  | SVM | C | 1.8738 |
|  |  | Gamma (γ) | 0.0811 |
|  | RF | Number of estimators | 80 |
|  |  | Max depth of trees | 5 |
|  |  | Min samples split | 5 |
|  |  | Min samples leaf | 15 |
|  |  | Bootstrap method | True |
