# Supplementary material for: Predicting Depression, Anxiety, and Their Comorbidity among Patients with Breast Cancer in China Using Machine Learning: A Multisite Cross-Sectional Study
Source: Depress Anxiety. 2024 Jun 21;2024:3923160. doi: 10.1155/2024/3923160 (PMC11918714; doi:10.1155/2024/3923160)
Supplement: Supplementary 3 — Lasso regulation profile (see Supplementary Figure 1–3). [file 3923160.f3.docx]

**Supplementary material S3**

**
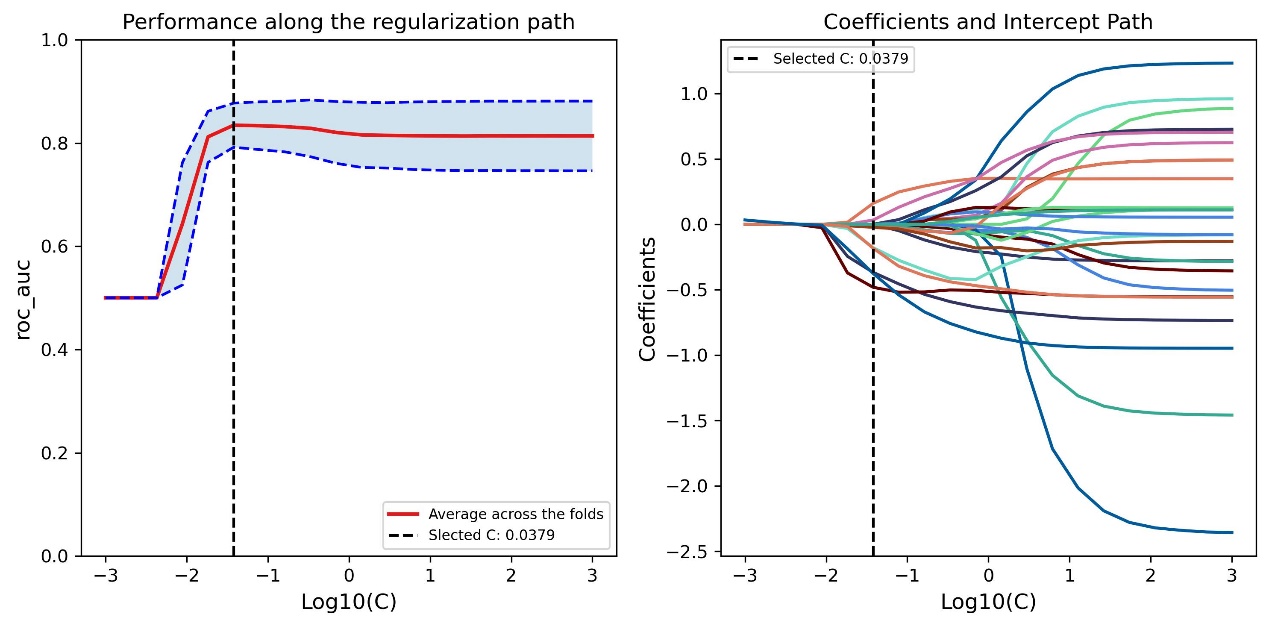
**

**Fig S1 Lasso regularization profile for depression**

**
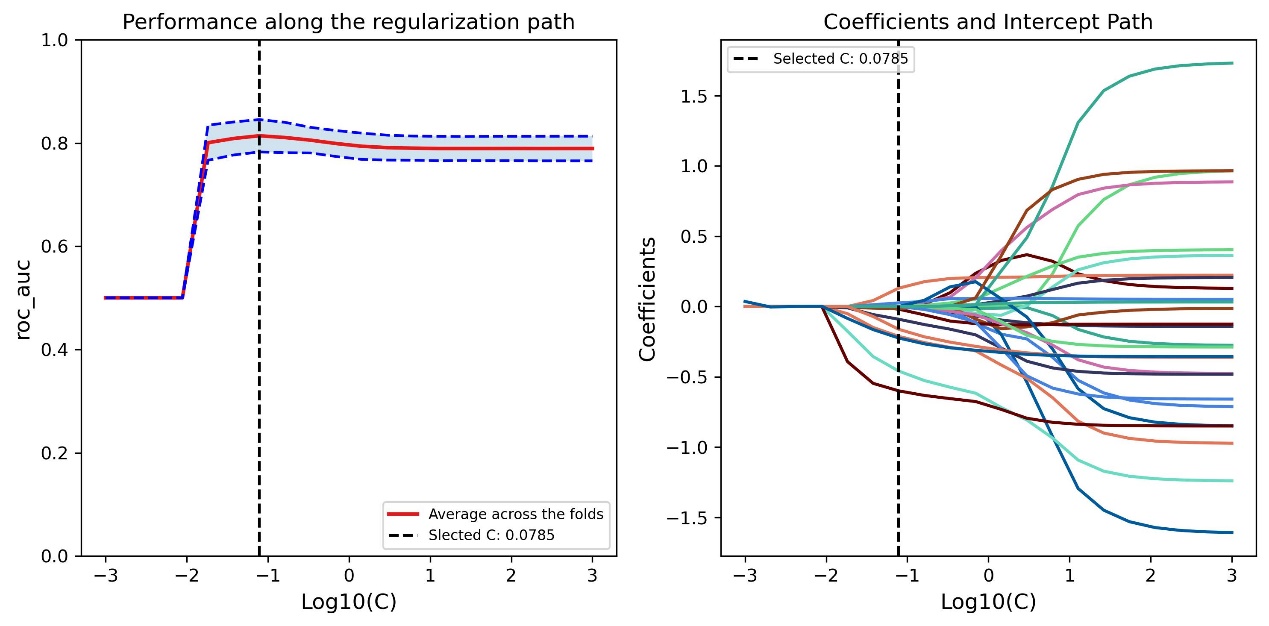
**

**Fig S2 Lasso regularization profile for Anxiety**

**
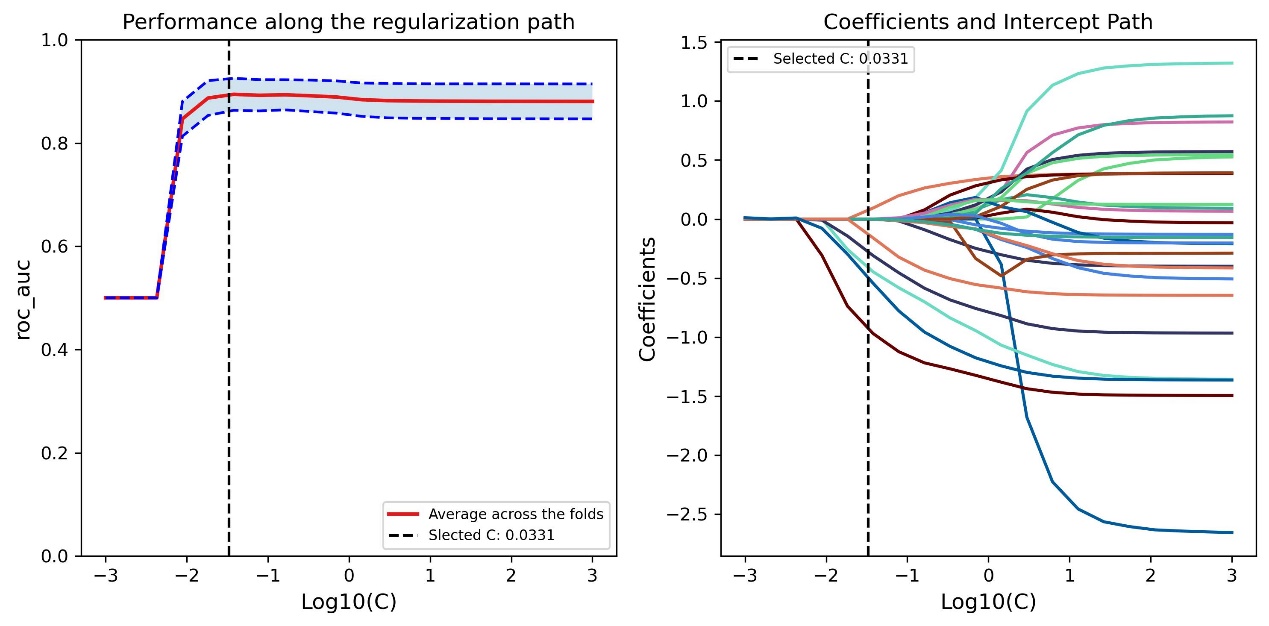
**

**Fig S3 Lasso regularization profile for CDA**
